# Synergistic Effect of Functionalized Nanokaolin Decorated MWCNTs on the Performance of Cellulose Acetate (CA) Membranes Spectacular

**DOI:** 10.3390/nano6040079

**Published:** 2016-04-22

**Authors:** Amina Afzal, Muhammad Shahid Rafique, Nadeem Iqbal, Asif Ali Qaiser, Abdul Waheed Anwar, Sadia Sagar Iqbal

**Affiliations:** 1 Physics Department, University of Engineering and Technology, Lahore 54000, Pakistan; a4amina@gmail.com (A.A.); shahidrafiq@uet.edu.pk (M.S.R.); wah_uet@yahoo.com (A.W.A.); 2 HR Materials, Quaid-e-Azam Industrial Estate, Lahore 54000, Pakistan; 3 Department of Polymer and Process Engineering, University of Engineering and Technology, Lahore 54000, Pakistan; aaqaiser@yahoo.com; 4 Department of Polymer Engineering and Technology, CEET, University of the Punjab, Lahore 54000, Pakistan; sadiasagariqbal.pu@gmail.com

**Keywords:** multiwalled carbon nanotubes, cellulose acetate, functionalized nanokaoline, membranes, thermal properties, water flux, salt rejection

## Abstract

In order to enhance salt rejection level and high pressure mechanical integrity, functionalized nanokaolin decorated multiwall carbon nanotubes (FNKM, 0–5 wt % loading) were incorporated into a cellulose acetate (CA) matrix using high temperature solution mixing methodology. Scanning electron microscopy (SEM), X-ray diffraction technique (XRD), thermo-gravimetric analyzer (TGA) and Fourier transform infrared spectrometer (FTIR) were used to characterize the prepared membranes. The obtained results revealed that with increasing FNKM concentration in the host polymeric matrix, composite membrane’s structural, functional, thermal, water permeation/flux and salt rejection characteristics were also modified accordingly. Percent enhancement in salt rejection was increased around threefold by adding 5 wt % FNKM in CA.

## 1. Introduction

A vast variety of polymeric membranes from micro porosity to dense films/fibers are employed for a broad range of applications *viz.* desalination, chemical separation/purification, medical applications, gas separation, *etc.* [1,2,3,4,5]. Some of the reasons behind the motivation of researchers throughout the world to develop polymeric membranes for set applications are easy to synthesize, geometrical adoptability, relatively cheaper technology for separation technologies, manageable pore size and density, good mechanical integrity, and comfort to install for targeted applications [6,7,8].

In order to synthesize membranes, multiple synthesis processes *viz.* thermally induced phase separation (TIPS), thermally assisted evaporation process (TAEP), *etc.* are used to generate porosity (micro to dense size) within the polymeric matrix (material) [9]. Polymer composite membranes are prepared to enhance thermal, mechanical, electrical, magnetic and chemical integrity [6,10]. These membranes are categorized as: (a) polymer–polymer blending membranes, (b) particle-filled polymeric membranes, and (c) fiber-filled polymeric membranes.

The interest to synthesized polymeric nanocomposite membranes (based on polymeric host matrix and nanoreinforcements) is generally increasing day by day [4,8,11]. Nanoreinforcement (filler/fiber type) has the advantages *i.e.*, large surface area, nanoscale interaction with the polymeric chains and very low weight percent loading required to acquire the desire characteristics of the prepared membranes compared to a macro or microreinforcement [2,8,12,13]. The drawbacks enclosed behind the nanoreinforcement usage are their agglomeration problem, inhomogeneous distribution within the host matrix, and weak interfacial bonding with the polymeric chains [12]. These problems can be overcome by proper functionalization of the incorporating nanoreinforcement. Usually an organic functional group is attached on the reinforcement surface that enhances filler to matrix compatibility as well as dispersibility. Among various developed methodologies/processes to functionalize nanoreinforcement, silane coupling agent (SCA) is one of the easiest and safer routes to actively attach functional group moiety on the surface of nanofiller. As in acidic treatment of nanofibers or carbon nanotubes (CNTs), aspect ratio suffers while in SCA treatment, and it does not happen [11,14,15].

Among the nanoreinforcement family, CNTs exist in the leading class due to its exceptional thermal, mechanical, electro-magnetic, optical and radiation absorption characteristics. Different types of nanofillers *viz.* nanoferrites, silica, silicon carbide, alumina, platinum, gold, *etc.* are employed on the surface of CNTs in order to introduce specific character for a particular application [15,16,17,18,19]. On one hand, pristine and functionalized multiwall carbon nanotubes (MWCNTs) with diverse ratios have been incorporated in variant polymeric membranes e.g., polyethersulfone (PES), polyamide (PA), polysulfone (PSf), cellulose acetate (CA) and alumina/PES hybrid membranes to tune/enhance their morphological and performance characteristics [5,15,20,21,22,23]. On the other hand, the toxicity hazards of MWCNTs cannot be avoided. *In vivo*/*vitro* studies revealed that MWCNTs can diversely affect human health causing injury to macrophages, blood coagulation, unspecific inflammation and cellular swelling if it exceeds the toxic dosage limit. Moreover, functionalized MWCNTs can easily cross cell wall and directly interact with DNA causing genotoxic [24,25]. The toxicity dosage value for MWCNT doped polymeric materials is 10 mg/mL (*in vitro*) [26]. Therefore, either the dosage of MWCNTs must be less than the toxic limit, or some other serious measures should be taken to cope with this adversity.

In the research presented, novel nanofiller (in multiple concentrations) have been introduced that has not only muddled through the toxicity hazards of MWCNTs but has also excellently improved the water permeation and salt rejection characteristics of CA membranes. The nanofiller FNKM comprise of functionalized nanokaolin (FNK) coated on MWCNTs. In our case, FNK to MWCNT ratio is 97:1 which means that 1g of FNKM has only 10mg MWCNTs. This nominal amount of MWCNTs is further covered by FNK consequently toxicity hazards of MWCNTs are negligible in our case. FNKM is used to incorporate into cellulose acetate (CA) matrix adopting solution mixing methodology. Thermally assisted evaporation process (TAEP) is adopted to synthesize the polymeric membranes. The effect of different concentrations of FNKM on the thermal, structural, functional, permeation and salt rejection characteristics of the developed membranes is studied herein.

## 2. Results and Discussion

### 2.1. SEM Analysis

**Figure 1:**
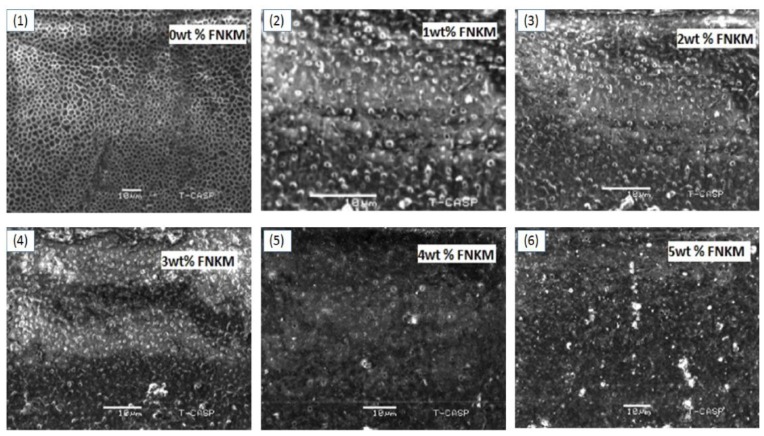
Scanning electron microscopy (SEM) images of cellulose acetate (CA) membranes with 0–5 wt % loading of FNKM (functionalized nanokaolin decorated multiwall carbon nanotubes).

**Figure 2:**
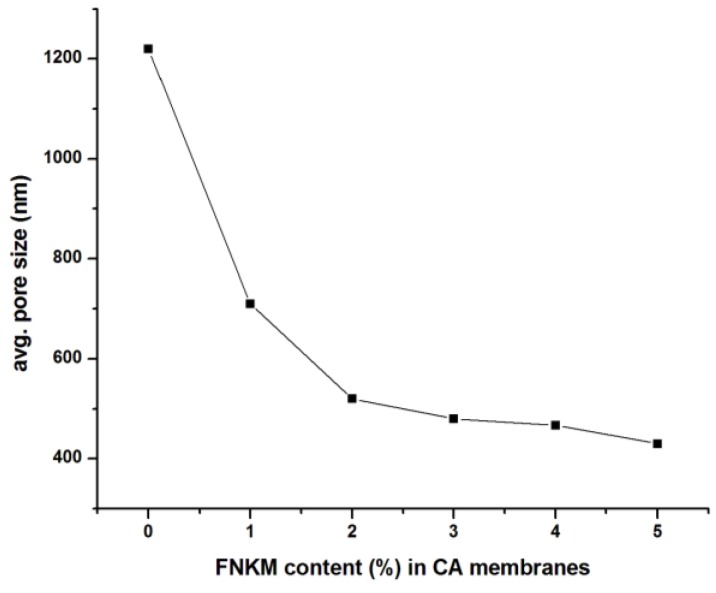
Correlation between FNKM content (%) loading in CA membranes and average (avg.) pore size.

Scanning electron microscopy (SEM) images of the synthesized polymeric membranes confirm the microporosity generation integrated throughout the polymeric films (Figure 1). The membranes prepared from the virgin polymer matrix, demonstrate homogenous pore network having average pore density and size equal to 250,000 pores/m^2^ and 1.22 μm, correspondingly. The generated pore geometry is observed mostly hexagonal and rarely spherically/ elliptical (Figure 1a,b). The adopted methodology, TAEP, used (above the boiling conditions of polymer solvent *i.e.*, acetone) to generate porosity, gives unique and homogenous pore structure/pattern, which is rarely observed in the contemporary data for CA microfiltration membranes [27]. Mostly, TAEP technique is used to synthesize nano/ultra/reverse osmosis (RO) membranes. The membrane solution preparation in acetone vapor state drives the thermal loads to generate microporosity within the polymeric films. When the solution is cast over the Teflon surface at 20 °C, the volatile components solvent/non-solvent evaporate rapidly, generating microporosity rather than nanoporosity in the meantime. The gradual FNKM addition in the polymeric matrix reduces both pore density and size of the developed membranes synergistically. The observed pore geometry for the composite membranes is mostly spherical and minimal elliptical (Figure 1c–g). The measured average pore sizes (using SEM images of the composite membranes 1–5 wt % loaded) are 710, 520, 480, 467 and 430 nm, correspondingly. Figure 2 indicates an exponential decay in pore size with increase in FNKM 0–5 wt % loading in the host CA matrix. It is attributed because the FNKM incorporation remarkably enhances thermal stability of the composite membranes and also develops interconnected network within the host matrix that resists thermal loads as well as the voids formation (as revealed through TGA curves). SEM images also illustrate insignificant FNKM agglomeration throughout the polymeric membranes, which means that proper filler functionalization and mixing methodology successfully homogenizes the nanofiller within the host polymeric matrix, therefore improving not only the compatibility concerns but also resolving dispersion issues synergistically.

### 2.2. FTIR Analysis

**Figure 3:**
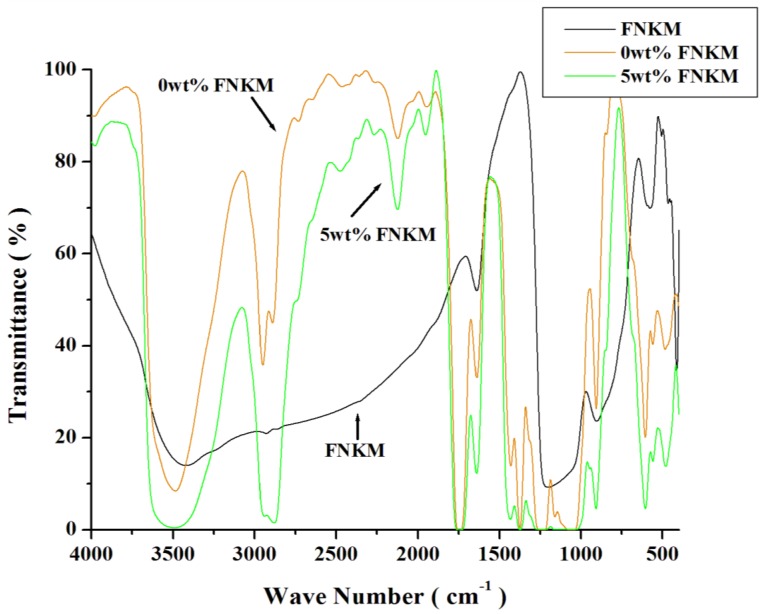
Fourier transform infrared spectrometer (FTIR) spectra of FNKM, 0 and 5 wt % FNKM loaded CA membranes.

The spectrum (Figure 3) of virgin CA, FNKM and 5 wt % of nanofiller loaded polymeric membranes demonstrates relative functional groups associated with the described molecules. The peaks at 3484 cm^−1^ (–OH stretching vibration), 2949/2890 cm^−1^ (–CH, –CH_2_, –CH_3_ stretching vibration), 2464 cm^−1^ (–OH stretching vibration of glycol molecule), 2122 cm^−1^ (C–C closed/benzene ring vibration), 1747 cm^−1^ (–C=O stretching mode), 1638 cm^−1^ (C=C stretching), 1430 cm^−1^ (–CH_2_ symmetric stretching), 1374 cm^−1^ (–C–C bending mode), 1230 cm^−1^ (–C–O in stretching mode), 1160 cm^−1^ (C–O–C bridge anti symmetric string), 1045 cm^−1^ (–C–O stretching mode), 946 cm^−1^ (carbonyl group) and 906 cm^−1^ (asymmetric ring C–C stretching) confirm the presence of all functional groups associated to CA chemical structures [28,29,30,31,32,33]. In Fourier transform infrared spectrometer (FTIR) spectrum of FNKM, peaks at 1638 cm^−1^ is C–C referring chiral structure of MWCNTs. The peaks at 1200 cm^−1^ (Si–O–CH_3_ vibration), 904 cm^−1^ (–CH_3_ attached with silicon), 505, 465 and 412 cm^−1^ (Si–O–) indicate silane moiety attachment on the surface of nanokaolin as well as MWCNTs. Silane functional groups developed a bridge between MWCNTs and nanokaolin surfaces. When being incorporated into CA, a second interlink develops between nanokaolin and the host polymeric chains due to the silane functional group presence [28,29,30,31,34,35]. Table 1 contains major functional groups and their transmittance frequency region.

Another interesting observation is that by incorporating FNKM into the virgin (having 0 wt % FNKM) polymer peak broadening and peak intensity enhancement are happened against all the responding functional groups. It is attributed to the three dimensional molecular environment variations due to the presence of FNKM. Consequently, the vibrational frequency band expands as compared to the pure polymeric state due to the affective presence of the functionalized nanoreinforcement [11]. The relative percent transmittance is also decreased by the addition of FNKM because polymeric chain mobility and dipole moment of the functional groups suppress with the addition of the reinforcement in the host polymeric matrix [18,33].

### 2.3. Thermo-Gravimetric/Differential Thermal Analyses (TGA/DTA)

**Figure 4:**
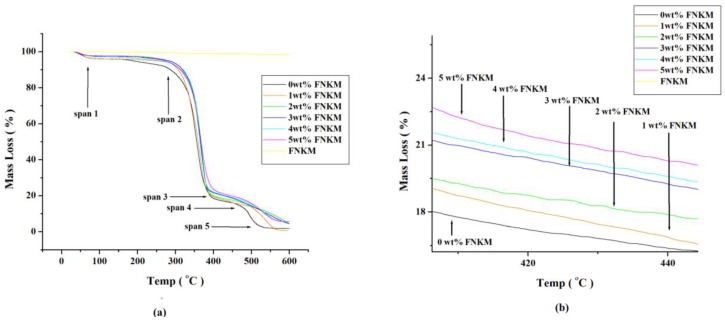
Thermo-gravimetric analyzer (TGA) curves of (**a**) FNKM and 0–5 wt % FNKM loaded CA membranes; (**b**) magnified image of span 4 at temperature rising rate of 10 °C/min in oxygen environment.

The presented thermal degradation graph of the synthesized CA formulations having different feedstock FNKM loadings shows five mass loss regimes (Figure 4a). In the first temperature span (0–82 °C), 4 mass% thermal decomposition is observed for all compositions that may be attributed due to the adsorbed moisture contents [36]. The second temperature region (83–285 °C) symbolizes insignificant mass loss for all polymeric systems. The third thermal premise (286–395 °C) reveals major thermal decomposition (77% mass loss) of the composite specimens. It is attributed due to the host polymeric chain scission and eventual charring [28,36]. This regime also depicts six mass percent thermal stability enhancements due to the nanofiller FNKM impregnation in the virgin polymeric matrix. Synergistic outcome of functionalized NK coated multiwall carbon nanotubes that are covalently attached with the host matrix (Figure 3) shows significant impact to delay thermal oxidation of the composite specimens. While CA matrix loaded with merely MWCNTs show almost the same thermal degradation as that of pristine CA membranes [16].

Homogeneously distributed functionalized nanofillers within the polymeric matrix develops an interlink network that offers remarkable resistance to the imposed thermal loads [28]. The fourth temperature expanse (395–475 °C) exhibits thermal stability improvement up to five percent (for 5 wt % FNKM loaded CA membranes) relative to the starting formulation (Figure 4b) attributed to the good distribution and exceptional thermal stability of the incorporated nanofillers [2]. The last temperature span (475–600 °C) illustrates char formation process completion (Figure 4b) [30]. Each thermal decomposition curve sets at a particular position (in the % mass loss against temperature graph) correspondingly to the FNKM concentration in CA composites. Thermal decomposition pattern of the synthesized nanofiller FNKM reveals negligible mass loss because both MWCNTs and nano-kaolin do not show thermal oxidation in the tested temperature regime.

**Figure 5:**
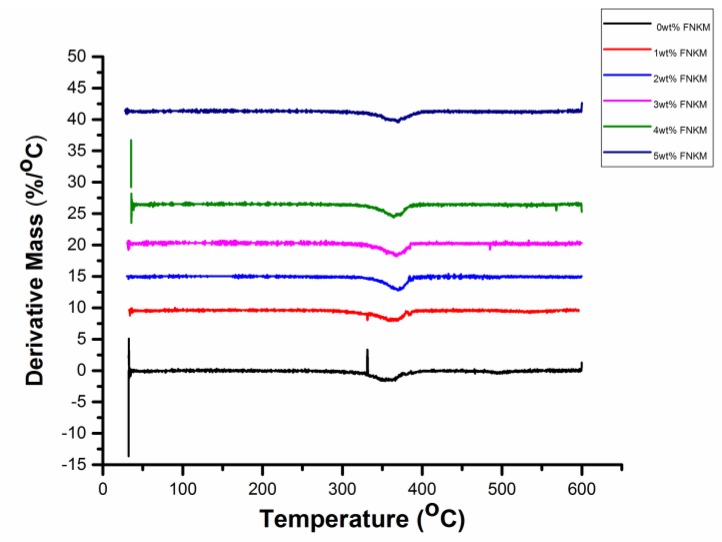
Differential thermal gravimetric (DTG) curves of 0–5 wt % loaded FNKM in CA membranes.

Differential thermal gravimetric analysis (Figure 5) of the prepared specimens demonstrates major peak variation across the temperature premises *viz.* 330 to 415 °C. It elaborates that major polymer degradation or melting is carried out across the aforesaid temperature range which is in accordance with the presented TGA graph. With increasing filler to matrix ratio, polymer degradation tends to delay due to excellent thermal stability and good mixing of FNKM within the host polymer matrix. Almost 30 °C delayed thermal decomposition is recorded for 5 wt % FNKM loaded CA membranes as compared to virgin one confirming its excellent compatibility with the host matrix owing to proper functionalization of the incorporated filler.

### 2.4. XRD Analysis

**Figure 6:**
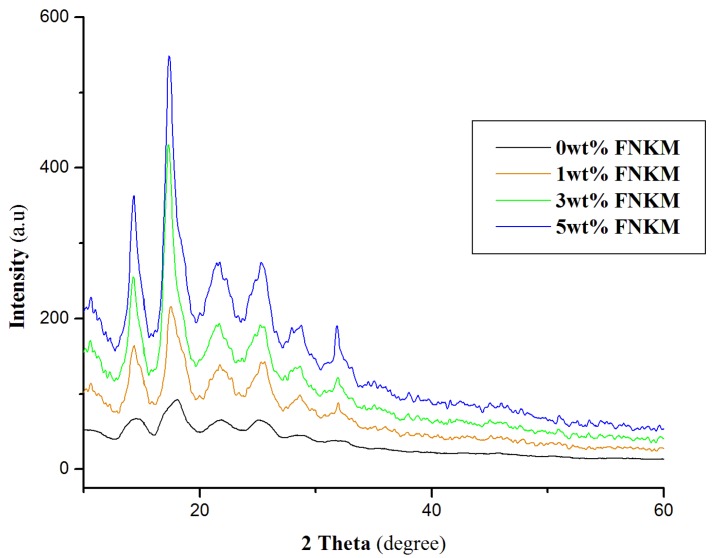
X-ray diffraction (XRD) pattern of 0, 1, 3, 5 wt % FNKM loaded CA membranes.

Figure 6 demonstrates the XRD pattern of 0, 1, 3 and 5 wt % FNKM loaded CA membranes. The peaks at 14, 17, 21 and 25 (2 Theta) confirm the polycrystalline nature of the virgin polymer matrix [37]. The addition of functional nano-reinforcement affects the crystallinity of the host matrix by decreasing full width half maximum (FWHM) of the obtained peaks. The maximum intensity peak at 17 (2 Theta) indicates 3.28 times peak narrowing by the 5 wt % impregnation of the nanofiller. The inherent peaks of nanokaolin and MWCNTs do not appear in CA nanocomposite membranes, which simulates the proper polymer encapsuling and distribution of the nanofiller within the premises of the host matrix. Agglomerated particles/nanofillers in a polymer matrix respond to the incoming X-rays while the homogenous filler dispersion on a nanoscale does not counter the incoming X-ray signal. Consequently, in the XRD pattern of the polymer nanocomposite, nanofillers do not exhibit their characteristic XRD pattern [19].

### 2.5. Membrane Performance

#### 2.5.1. Applied Pressure *vs.* FNKM Content (%)

**Figure 7:**
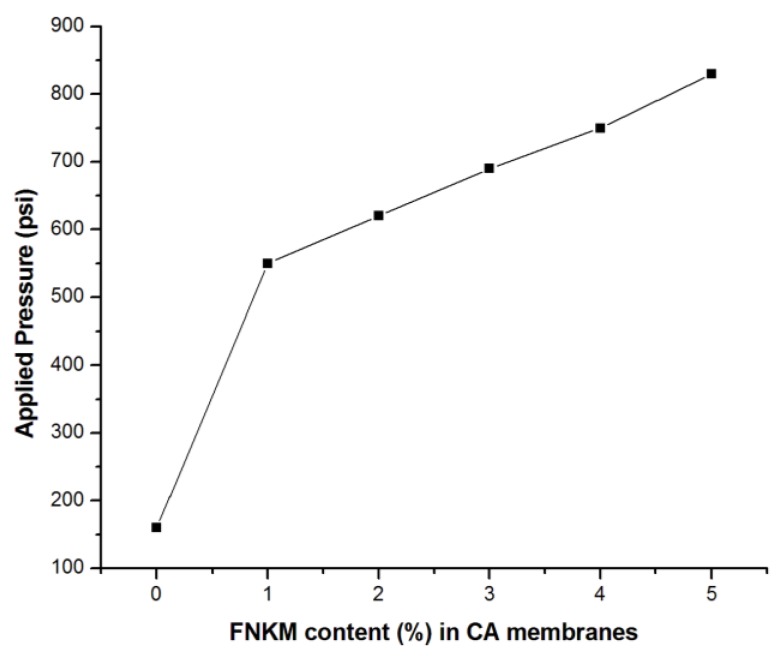
Relationship between FNKM loading content and applied pressure.

Figure 7 explores the applied pressure profile at which the membranes start permeation. Deionized water is used to study the pressure profile of the synthesized membranes. The accumulative data reveals that with increasing FNKM wt % loading in the host polymer matrix, the required applied pressure also enhances gradually. Prior studies divulged that with variant wt % MWCNT loaded CA membranes initiate permeation within the applied pressure range of 60–508 psi [15,16,38]. In the presented research, however, the virgin polymer matrix membrane starts permeation at 160 psi while the membranes having FNKM loading of 1–5 wt % begin permeation in the pressure range 550–830 psi, correspondingly. This noteworthy uplift of applied pressure is a synergistic contribution of FNK along with MWCNTs. The applied pressure profile for the composite membranes indicates that the synthesized membrane family belongs to nanofiltration or RO membranes, which is a contradiction with the SEM images (Figure 1) [38]. SEM images indicate that the fabricated polymeric membranes have micro-porosity that can be employed over microfiltration processes, while the applied pressure profile for the composite membranes indicates the membranes category *viz.* nanofiltration or RO membranes. The required permeation pressure for RO and nanofiltration membranes is 300 and 400 psi, accordingly. This occurred because the observed microporosity (710, 520, 480, 467 and 430nm) with increasing loading concentration of FNKM) may be a dead-end rather than right thorough. The contemporary data reveals that TAEP is used to develop nanofiltration or RO membranes rather than microfiltration membranes [9]. The experimental applied pressure profile against the tested profiles endorsed the above mentioned statement. Another reason to enhance the applied pressure value is that, with increasing nanofiller loading in the host polymer matrix, both the pore density and size diminish accordingly [8]. The synthesized nanocomposite membranes demonstrate remarkable pore size reduction and mechanical integrity enhancement synergistically, even at a pressure of 830 psi. The close observation of membranes after permeation study reveals that there is no macro-crack or pore existing on the surface of the tested membranes.

#### 2.5.2. Flux *vs.* FNKM Content (%) in CA Membranes

**Figure 8:**
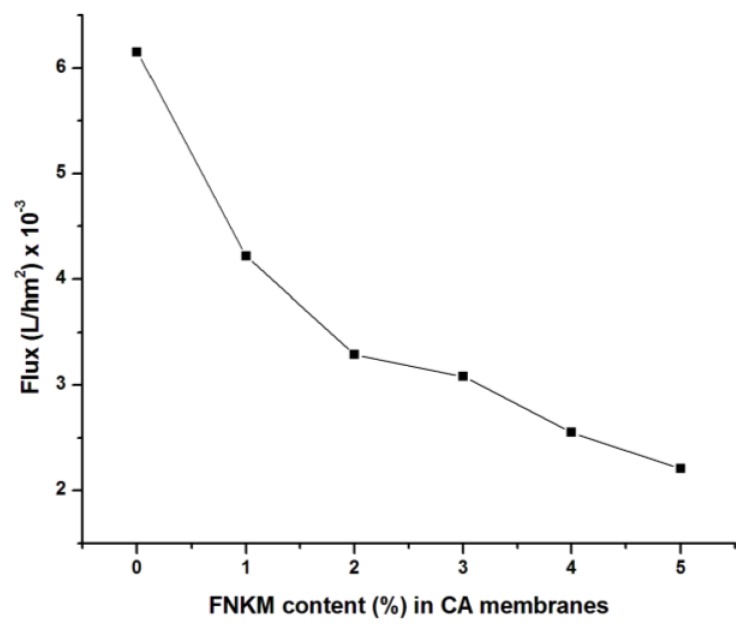
Correlation between FNKM loading contents and flux.

Figure 8 demonstrates that with increasing nanofiller to matrix ratio, DI water flux decreases in the meantime. The reason behind the experimental decay of flux resides with the decrease of pore size as well as density by increasing FNKM loading in the host polymer matrix.

#### 2.5.3. Salt Rejection *vs.* FNKM Content (%) in CA Membranes

**Figure 9:**
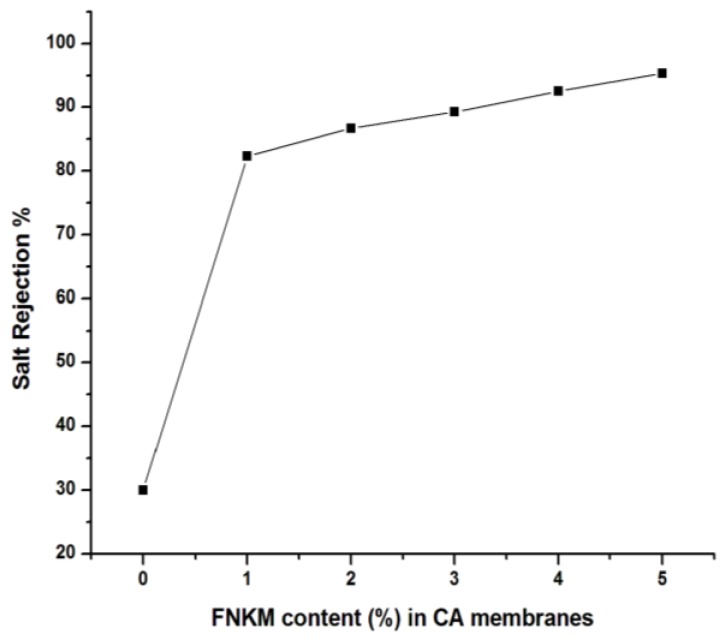
Correlation between FNKM loading content and salt rejection.

Figure 9 displays the practical study of percent salt rejection by the synthesized membranes. It is also conducted to explore its worth for practical applications *viz.* desalination. As the pore size/density decreases, salt rejection level of the synthesized membranes improve tremendously and consequently attains the value of 95.35% (5 wt % FNKM loaded membrane) as compared to 30% salt rejection (0 wt % FNKM loaded membrane). Since 1 wt % FNKM loaded CA membranes contain 0.01 wt % MWCNTs, which offer 82.3% salt rejection that is strikingly improved up to ~2.5 times compared to a previously reported 34% salt rejection capability of CA membranes loaded with the same MWCNT content [15]. Similarly, 5 wt % FNKM loaded membranes exhibited 95.35% NaCl rejection, which is again ~20% boosted up in comparison to the CA/MWCNTs with the same loading (0.05 wt %) [16]. This noticeable improvement in salt rejection is clearly attributed to the synergistic effect of FNK that is being decorated on the MWCNTs. The lower water flux and high salt rejection of the nanocomposite membranes are prescribed to enhance the pore density or applied pressure, in order to counter the low permeation rate. Membrane performance has been summarized in Table 2.

#### 2.5.4. Time *vs.* FNKM Content (%) in CA Membranes

**Figure 10:**
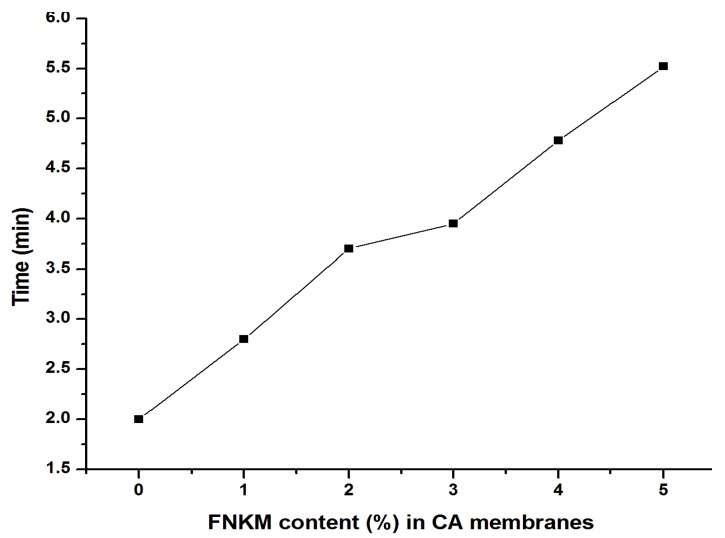
Correlation between FNKM loading content and time taken to collect 100mL permeate.

Figure 10 exhibits the time (min) taken by synthesized CA membranes against a certain applied pressure for a 100 mL amount collected as the permeate of DI water. The graph indicates an increasing trend between time and FNKM loading in the prepared membranes. This is due to decrement in pore density and size as depicted by SEM images in (Figure 1). The presence of functionalized MWCNTs resists the void formation by interconnecting the host polymer matrix chains and eventually pore size and density decrease with increasing filler to matrix ratio.

## 3. Experimentation

### 3.1. Materials and Methods

#### 3.1.1. Materials

Cellulose acetate (CA, M_w_ ~50,000, acetyl content 39.7 wt %, water content ≤ 3.0%), polyethylene glycol (PEG, M_w_ ~6000), acetone (purity ≥ 99.8%), sodium chloride (NaCl, purity ≥ 99.5%), MWCNTs (diameter 20–25 nm, length 1.5–2 μm and C-purity ≥ 98%), kaolin (NK, analytical grade, pH ~3.5–5), 3-aminopropyl trimethoxysilane (APTMS, purity ≥ 97%), and certimonium bromide (CTAB, analytical grade) were purchased from Sigma Aldrich (GmbH, Taufkirchen, Germany). All chemicals are used as received without any further treatment.

#### 3.1.2. Fabrication Method

FNKM was synthesized in ultrasonication bath at frequency → 60 KHz and temperature → 85 °C in deionized (DI) water for 30 h. APTMS and CTAB are used to interlink/capping MWCNTs and NK and also with the surface of the host matrix. The ratio of the NK to MWCNTs to APTMS to CTAB is 97:1:1:1, respectively.

The TAEP process was used to synthesize polymeric membranes in which water acts as a non-solvent and acetone as a solvent for CA. In all polymeric solutions, 50 ml acetone was used while the quantities of CA, PEG and DI water were selected with respect to the acetone extent. The weight ratio of CA:PEG:Acetone:DI water was kept at 7.2:2.8:100:3 as detailed in Table 3. The solution was vigorously stirred at 1200 rpm and 70 °C for 24 h in a glass sealed bottle. This practice is used to homogenize the polymer matrix within the solvent. After that, the membrane solution was kept at room temperature for 24 h and then cast on the Teflon sheet using an adjustable film applicator (Sheen 1117/150, Sheen Instruments, Cambridge, UK). Six different types of membranes based on FNKM loading (0–5 wt % with respect to CA) were prepared using the above mentioned methodology to elucidate the effect of functionalized nanoreinforcement on the thermal, structural, functional, permeation and salt rejection characteristics of CA membranes. The derived membranes were put into ice water at 4 °C, and then the temperature was gradually increased on the hot plate up to maximum 90 °C for homogeneous and controlled evaporation of the impregnated DI water included in the polymeric membranes. The thickness of the prepared membranes was in the range of 40–60 μm.

### 3.2. Characterization

**Figure 11:**
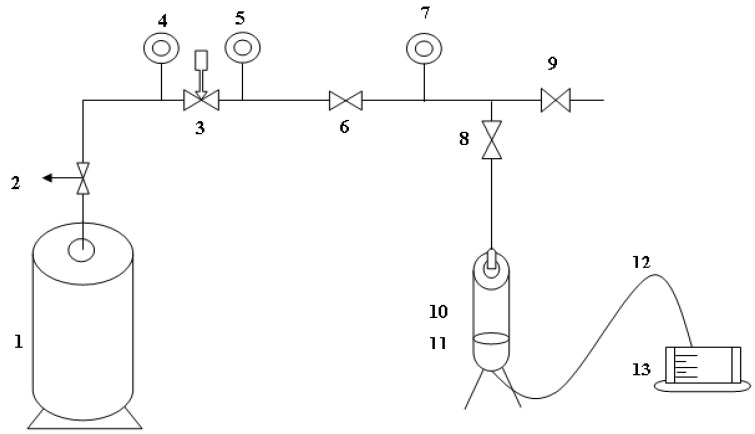
Schematic for experimental setup used for permeation and salt rejection tests. The image includes: N_2_ gas cylinder (**1**); opening valve (**2**); pressure regulator (**3**); pressure gauges (**4**, **5** and **7**); gas release valve (**6**); gas valve towards cylinder (**8**); gas purge to outside (**9**); permeation module (**10**); membrane holder (**11**); permeation outlet (**12**); and volumetric container (**13**).

For each formulation, five samples were used for tests and characterization. Scanning electron microscopy (SEM, JSM 6490A JEOL, Tokyo, Japan) was used to observe the surface texture of the prepared membranes. In addition, 2 nm gold coating on the polymeric membranes specimen was conducted before characterization. SPI-module ion sputter coater was used in this regard. Thermal oxidation study was conducted on TGA (TGA50 Shimadzu, Kyoto, Japan). The operational temperature range of TGA was 30–600 °C and the temperature ramp was 10 °C/min in oxygen environment. For TGA, 10 mg sample for each test was exploited. Functional group determination was carried out on an FTIR spectrometer (FTIR Prestige 21, Tokyo, Japan), in attenuated total reflectance (ATR) mode by using ZnSe (Zinc Selenium) crystal as the background). The operating wave number range was 400–4000 cm^−1^. Before FTIR analysis, the formulated samples were washed with DI water and dried in a vacuum oven at 70 °C for 1 h. The structural study of the prepared membranes was characterized using an X’PERT X-rays diffractometer (Almelo, the Netherlands) using Cu Kα radiation of wavelength 1.506 Å The diffraction pattern was obtained within a 2θ range of 10–60° at a scan rate of 0.15°/s. 2 cm^2^ area of the membranes that were utilized for XRD study. The synthesized membranes’ efficiency regarding DI water permeation flux and salt rejection was carried out on a domestically fabricated nanofiltration (dead-end) module. Nitrogen gas was used to exert tuneable pressure on the water/solution to permeate through the membrane. The schematic for experimental setup used for permeation and salt rejection tests is represented in Figure 11. In addition, 1000 PPM (part per million) solution of NaCl was prepared in DI water to make a salt solution to validate salt rejection performance of the developed membranes. PPM of the developed solution before and after permeation/rejection study was carried out using TDS (TDS-161002) meter (Mokena, IL, USA). Here TDS stands for total dissolved solid which are present in solution. Membranes were dipped in DI water for 12 h before permeation and rejection study. DI water permeation flux was determined using Equation (1):

*J* = (*Q*/*t*) × *A* (1)

where *J* is DI water permeation flux (measured in L/hm^2^), *Q* is the amount of permeate (in L), *t* is the time (in min) to fill a definite volume, and *A* is the area (in m^2^). The affected diameter of the membranes was 2 inches used in a dead-end nanofiltration module on which pressure (in psi) was applied. Salt rejection of NaCl was calculated using Equation (2)

*R* (%) = (1 – *C*_p_/*C*_f_) × 100 (2)

where *R* is representing salt retention rate (in percentage), *C*_p_ and *C*_f_ (both in PPM) are the salt concentration in permeate and feed, respectively. DI water permeation flux and salt rejection was determined in quadruplicate for each formulated membrane, and the average is reported.

## 4. Conclusions

The present study revealed the synergistic impact of nanokaolin (NK) decorated MWCNT (0–5 wt %) loadings on the morphological, structural, permeation and salt rejection characteristics of CA composite membranes. The TAEP process was used to synthesize the polymeric membranes. SEM study elucidated remarkable pore size reduction by increasing the nanoreinforcement to matrix ratio. The generated pore geometry in the polymeric membranes was also influenced (hexagonal to spherical shaped) by the addition of FNKM. The functionalization of FNKM, functional groups associated with CA, and the presence of FNKM in the host matrix was confirmed by FTIR study. XRD patterns of the composite membranes delineate the crystallinity improvement of CA membranes with the FNKM incorporation. Delayed thermal decomposition and higher thermal stability of FNKM loaded membranes as compared to virgin CA membranes are observed in TGA. With increasing filler to matrix ratio, the required applied gas pressure to start permeation was increased correspondingly, and, on the other hand, flux through the polymer membranes was gradually reduced by 64.23% due to pore size/density reduction of the prepared membranes. Excellent improvement in salt rejection level was observed by increasing the FNKM loading in CA. In addition, 95% salt rejection was obtained for 5 wt % loaded membranes as compared to virgin CA membrane (30% salt rejection). It revealed that the membranes with 4–5 wt % can be employed for the desalination purposes. The conducted research gives the direction to utilize this novel filler FNKM to synthesize nanocomposite membranes for RO and pervaporation purposes. Complete optimization of the synthesized membranes for practical applications required dynamical mechanical thermal analysis (DMTA), molecular weight cut off (MWCO), membrane thickness *vs.* permeation, NK to MWCNT ratio, and toxicity hazards, *etc.*, which are in process.

## Figures and Tables

**Table 1:** Containing major functional groups and their transmittance frequency region.

Sample Name	Wave Number (cm^−1^)	Chemical Bond	References
0 and 5 wt % FNKM loaded CA membrane	3484	Stretching vibration of –OH exist in CA	[30,31]
	2949	Stretching vibration of –CH, –CH_2_, –CH_3_	[30]
	2890	Stretching vibration of –CH, –CH_2_, –CH_3_	[30]
	2464	Stretching vibration of –OH , evident of glycol	[33]
	2122	Stretching vibration of C≡C	[33]
	1747	Stretching vibration of carboxyl group of CA, C=0, Strong peak due to stretching mode of ester group	[30,32]
	1638	Strongly bonded –OH group in the matrix	[32]
	1430	–CH_2_ symmetric, or –CH_3_ asymmetric stretching mode	[30]
	1374	C–C bond stretching	[33]
	1230	C–O bond, contributed by acetate group	[29,31]
	1160	C–O–C bridge anti-symmetric stretching	[28,31]
	1045	C–O Stretching mode; contributed by acetate group	[29]
	906	Asymmetric ring stretching mode	[30]
5 wt % FNKM loaded membrane	2266	Si–O–CH_3_ molecules attached with FNKM	[35]
	1255	C–O stretch bond	[34]
FNKM	3420	Si–CH_3_ stretching mode	[35]
	1638	C–C chiral structure of MWCNTs	[34]
	1200	Si–O–CH_3_ vibration	[34,35]
	904	–CH_3_ attached with silicon	[28,29,30]
	505	Si–O– silicon moiety attachment	[30,31,35]
	465	Si–O– silicon moiety attachment	[30,31,35]
	412	Si–O– silicon moiety attachment	[30,31,35]

**Table 2:** Cumulatively expressing performance of FNKM loaded CA membranes.

FNKM Content (%)	Applied Pressure (psi)	Flux × 10^−3^ (L/hm^2^)	Salt Rejection (%)	Salt Passage (%)	Time (min)
0	160	6.15	30	70	2
1	550	4.22	82.3	17.7	2.89
2	620	3.29	86.7	13.3	3.7
3	690	3.08	89.25	10.75	3.95
4	750	2.55	92.5	7.5	4.78
5	830	2.20	95.35	4.65	5.52

**Table 3:** Compositions of the prepared solution used in casting CA/PEG/FNKM composite membranes. PEG: Polyethylene glycol.

Membrane	Solvent	Matrix Polymer	Additive
FNKM wt %	Acetones:CA/PEG (*v*/*w*)	CA:PEG (*w/w*)	FNKM:(CA/PEG)
CA/PEG/FNKM 0 wt %	10:1	72:27	0%
CA/PEG/FNKM 1 wt %	10:1	72:27	1%
CA/PEG/FNKM 2 wt %	10:1	72:27	2%
CA/PEG/FNKM 3 wt %	10:1	72:27	3%
CA/PEG/FNKM 4 wt %	10:1	72:27	4%
CA/PEG/FNKM 5 wt %	10:1	72:27	5%

## References

[1] Ashraf M.A., Maah M.J., Qureshi A.K., Gharibrez M., Yusoff I. (2013). Synthetic polymer composite membrane for the desalination of saline water. Desalin. Water Treat..

[2] Naghsh M., Sadeghi M., Moheb A., Chenar M.P., Mohagheghian M. (2012). Separation of ethylene/ethane and propylene/propane by cellulose acetate–silica nanocomposite membranes. J. Membr. Sci..

[3] Idris A., Yet L.K. (2006). The effect of different molecular weight PEG additives on cellulose acetate asymmetric dialysis membrane performance. J. Membr. Sci..

[4] Li J., Wang S., Nagai K., Nakagawa T., Mau A.W.-H. (1998). Effect of polyethyleneglycol (PEG) on gas permeabilities and permselectivities in its cellulose acetate (CA) blend membranes. J. Membr. Sci..

[5] Moghadassi A.R., Rajabi Z., Hosseini S.M., Mohammadi M. (2014). Fabrication and modification of cellulose acetate based mixed matrix membrane: Gas separation and physical properties. J. Ind. Eng. Chem..

[6] Nam B.-U., Min K.-D., Son Y. (2015). Investigation of the nanostructure, thermal stability, and mechanical properties of polylactic acid/cellulose acetate butyrate/clay nanocomposites. Mater. Lett..

[7] Filho G.R., Monteiro D.S., da Silva Meireles C. (2008). Synthesis and characterization of cellulose acetate produced from recycled newspaper. Carbohydr. Polym..

[8] Rahimpour A., Jahanshahi M., Mollahosseini A., Rajaeian B. (2012). Structural and performance properties of UV-assisted TiO2 deposited nano-composite PVDF/SPES membranes. Desalination.

[9] Van de Witte P., Dijkstra P.J., van den Berg J.W.A., Feijen J. (1996). Phase separation processes in polymer solutions in relation to membrane formation. J. Membr. Sci..

[10] Abdel-Naby A.S., Al-Ghamdi A.A. (2014). Chemical modification of cellulose acetate by *N*-(phenyl amino) maleimides: Characterization and properties. Int. J. Biol. Macromol..

[11] Yuwawech K., Wootthikanokkhan J., Tanpichai S. (2015). Enhancement of thermal, mechanical and barrier properties of EVA solar cell encapsulating films by reinforcing with esterified cellulose nanofibers. Polym. Test..

[12] Abedini R., Mahmoud Mousavi S.M., Aminzadeh R. (2011). A novel cellulose acetate (CA) membrane using TiO2 nanoparticles: Preparation, characterization and permeation study. Desalination.

[13] Zodrow K., Brunet L., Mahendra S., Li D., Zhang A., Li Q., Alvarez P.J. (2009). Polysulfone ultrafiltration membranes impregnated with silver nanoparticles show improved biofouling resistance and virus removal. J. Water Res..

[14] Rahimpour A., Jahanshahi M., Khalili S., Mollahosseini A., Zirepour A., Rajaeian B. (2012). Novel functionalized carbon nanotubes for improving the surface properties and performance of polyethersulfone (PES) membrane. Desalination.

[15] Badawi N.E., Ramadan A.R., Esawi A.M.K., El-Morsi M. (2014). Novel carbon nanotube–cellulose acetate nanocomposite membranes for water filtration applications. Desalination.

[16] Nezam El-Din L.A., El-Gendi A., Ismail N., Abed K.A., Ahmed A.I. (2015). Evaluation of cellulose acetate membrane with carbon nanotubes additives. J. Ind. Eng. Chem..

[17] Favvas E.P., Nitodas S.F., Stefopoulos A.A., Papageorgiou S.K., Stefanopoulos K.L., Mitropoulos A.C. (2014). High purity multi-walled carbon nanotubes: Preparation, characterization and performance as filler materials in co-polyimide hollow fiber membranes. Sep. Purif. Technol..

[18] Luo Y., Wang S., Shen M., Qi R., Fanga Y., Guo R., Cai H., Cao X., Tomás H., Zhu M. (2013). Carbon nanotube-incorporated multilayered cellulose acetate nanofibers for tissue engineering applications. Carbohydr. Polym..

[19] Ahmad A.L., Jawad Z.A., Low S.C., Zein S.H.S. (2014). A cellulose acetate/multi-walled carbon nanotube mixed matrix membrane for CO_2_/N_2_ separation. J. Membr. Sci..

[20] Celik E., Park H., Choi H., Choi H. (2011). Carbon nanotube blended polyethersulfone membranes for fouling control in water treatment. Water Res..

[21] Choi J.-H., Jegal J., Kim W.-N. (2006). Fabrication and characterization of multi-walled carbon nanotubes/polymer blend membranes. J. Membr. Sci..

[22] Feng Y., Wang K., Davies C.H.J., Wang H. (2015). Carbon Nanotube/Alumina/Polyethersulfone Hybrid Hollow Fiber Membranes with Enhanced Mechanical and Anti-Fouling Properties. Nanomaterials.

[23] Shawky H.A., Chae S.-R., Lin S., Wiesner M.R. (2011). Synthesis and characterization of a carbon nanotube/polymer nanocomposite membrane for water treatment. Desalination.

[24] Awasthi K.K., John P.J., Awasthi A., Awasthi K. (2013). Multi walled carbon nano tubes induced hepatotoxicity in Swiss albino mice. Micron.

[25] Lucia U.C., Delia C., Aureliano C., Maria F.A., Raffaele M., Buresti G., Casciardi S., Iavicoli S. (2015). Cytotoxic, Genotoxic and Proinflammatory Response of Human Bronchial Cells to Pristine and Functionalized MWCNTs. Mater. Today Proc..

[26] Firme C.P., Bandaru P.R. (2010). Toxicity issues in the application of carbon nanotubes to biological systems. Nanomedicine Nanotechnol. Biol. Med..

[27] Mahdavi H., Shahalizade T. (2015). Preparation, characterization and performance study of cellulose acetate membranes modified by aliphatic hyper branched polyester. J. Membr. Sci..

[28] De Moraes A.C., Andrade P.F., de Faria A.F., Simões M.B., Salomão F.C.C.S., Barros E.B., do Carmo Gonçalves M., Alves O.L. (2015). Fabrication of transparent and ultraviolet shielding composite films based on graphene oxide and cellulose acetate. Carbohydr. Polym..

[29] Kee C.M., Idris A. (2010). Permeability performance of different molecular weight cellulose acetate hemodialysis membrane. Sep. Purif. Technol..

[30] John A., Chen Y., Kim J. (2012). Synthesis and characterization of cellulose acetate–calcium carbonate hybrid nanocomposite. Compos. Part B.

[31] Worthley C.H., Constantopoulos K.T., Ginic-Markovic M., Pillar R.J., Matisons J.G., Clarke S. (2011). Surface modification of commercial cellulose acetate membranes using surface-initiated polymerization of 2-hydroxyethyl methacrylate to improve membrane surface biofouling resistance. J. Membr. Sci..

[32] Rathore B.S., Sharma G., Pathania D., Gupta V.K. (2014). Synthesis, characterization and antibacterial activity of cellulose acetate–tin (IV) phosphate nanocomposite. Carbohydr. Polym..

[33] Zafar M., Ali M., Khan S.M., Jamil T., Butt M.T.Z.B. (2012). Effect of additives on the properties and performance of cellulose acetate derivative membranes in the separation of isopropanol/water mixtures. Desalination.

[34] Bose S., Das C. (2015). Sawdust: From wood waste to pore-former in the fabrication of ceramic membrane. Ceram. Int..

[35] Ghouil B., Harabi A., Bouzerara F., Boudaira B., Guechi A., Demir M.M., Figoli A. (2015). Development and characterization of tubular composite ceramic membranes using natural alumino-silicates for microfiltration applications. Mater. Charact..

[36] Ahmad A., Waheed S., Khan S.M., e-Gul S., Shafiq M., Farooq M., Sanaullah K., Jamil T. (2015). Effect of silica on the properties of cellulose acetate/polyethylene glycol membranes for reverse osmosis. Desalination.

[37] Wu C.-S. (2012). Characterization of cellulose acetate-reinforced aliphatic–aromatic copolyester composites. Carbohydr. Polym..

[38] Sabir A., Shafiq M., Islam A., Sarwar A., Dilshad M.R., Shafeeq A., Butt M.T.Z., Jamil T. (2015). Fabrication of tethered carbon nanotubes in cellulose acetate/polyethylene glycol-400 composite membranes for reverse osmosis. Carbohydr. Polym..

